# Bioactive-Loaded Zein Nanofiber Coatings for Improving Microbiological and Oxidative Quality, and Extending the Shelf Life of Chicken Meatballs

**DOI:** 10.1021/acsomega.6c04484

**Published:** 2026-07-21

**Authors:** Nazan Kutlu, Raciye Meral

**Affiliations:** Department of Food Engineering, Faculty of Engineering, 53000 Van Yüzüncü Yıl University, Van 65080, Türkiye

## Abstract

In the present study, zein (ZN)-based nanofibers incorporating flaxseed oil (FSO), curcumin (CUR), and gallic acid (GA) were fabricated, systematically characterized, and investigated as active edible coatings for chicken meatballs stored under refrigerated conditions (4 ± 2 °C). Three different nanofiber formulations were developed: curcumin-loaded zein nanofibers (ZNC), zein nanofibers co-loaded with flaxseed oil and curcumin (ZNFC), and zein nanofibers simultaneously incorporating flaxseed oil, curcumin, and gallic acid (ZNFCG). The produced nanofibers exhibited uniform morphologies with average fiber diameters ranging from 135.93 to 338.55 nm and demonstrated high encapsulation efficiencies (97.13–98.03%). Antimicrobial activity against selected foodborne pathogens was confirmed, as evidenced by inhibition zone diameters of 14–16 mm and minimum inhibitory concentration values as low as 0.05 mg/mL. In addition, in vitro release profiles, total phenolic content, and antioxidant capacity assessed by the DPPH assay were evaluated. Following the application of nanofiber coatings, chicken meatballs were monitored throughout refrigerated storage until the total mesophilic aerobic bacteria (TMAB) count reached the microbiological acceptability threshold of 6 log CFU/g. Changes in microbiological quality, including TMAB, total psychrophilic bacteria, and total yeast and mold counts, as well as oxidative stability parameters such as thiobarbituric acid (TBA) values and protein carbonyl content, were systematically analyzed during storage. On the third day of storage, the TMAB count in the uncoated control samples reached 6.11 log CFU/g, whereas ZNFCG-coated samples maintained significantly lower levels (3.40 log CFU/g). Overall, the application of zein-based nanofiber coatings effectively suppressed microbial proliferation and oxidative degradation, with the ZNFCG formulation exhibiting the most pronounced protective effect. These results indicate that multibioactive zein nanofiber coatings, particularly the ZNFCG formulation, have promising potential as a laboratory-scale active coating system for improving the refrigerated storage stability of chicken meatballs. However, further food-contact safety, migration, mechanical integrity, adhesion, and refrigerated-release studies are required before practical or industrial application.

## Graphical abstract

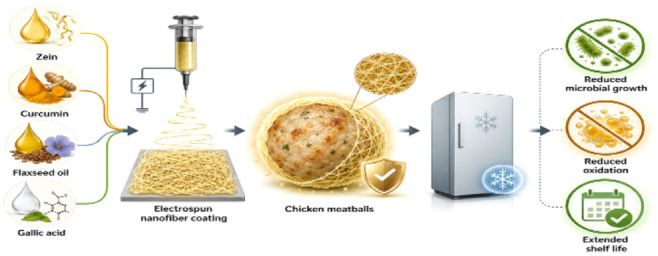

## 1 Introduction

Meat and meat products constitute an essential component of human nutrition, owing to their high-quality protein and valuable mineral content. Among these, white meat products are particularly favored due to their rich protein composition, comparatively low fat content, and greater economic accessibility.^1^ Compared to red meat, chicken meat contains higher levels of unsaturated fatty acids, particularly oleic and linoleic acids, and constitutes an important nutritional source owing to its low carbohydrate content and its richness in B-group vitamins, especially niacin. Given that a substantial proportion of the daily protein requirement is recommended to be met through animal-based foods, chicken meat, with an approximate protein content of 20–22% and lower cholesterol levels than red meat, emerges as a valuable source of animal protein in human nutrition.^2^

The principal factors contributing to quality deterioration and shelf life reduction in meat and meat products are oxidative degradation and microbial contamination. Oxidative reactions negatively influence sensory attributes, including taste, texture, color, and aroma, while simultaneously facilitating the formation of potentially harmful compounds that may pose risks to human health. In meat products, oxidative processes affect not only the lipid fraction but also protein structures, giving rise to structural modifications, such as amino acid side-chain alterations, peptide bond cleavage, protein aggregation, and cross-linking. These changes, collectively defined as protein oxidation, lead to a pronounced deterioration in nutritional value and sensory quality, thereby significantly reducing the shelf life of meat products.^3^ Consequently, the incorporation of antioxidant compounds into food systems is regarded as an effective strategy for mitigating oxidative reactions and maintaining the product quality.^4^

Microbial spoilage constitutes another critical factor compromising the quality and safety of meat and meat products as the presence of pathogenic microorganisms may give rise to serious public health concerns. Food products are vulnerable to microbial contamination at various stages, including production, processing, transportation, and preparation, and the proliferation of microorganisms under unsuitable conditions can lead to adverse food safety outcomes.^5^ In particular, contamination with pathogenic microorganisms such as *Salmonella* spp., *Staphylococcus aureus*, and *Listeria monocytogenes* can lead to severe infections that may potentially be fatal.^6^ Accordingly, growing attention has been focused on the utilization of natural antimicrobial compounds derived from plant sources, such as rosemary, grape seed, green tea, and pomegranate peel, in meat products.^7^ These plant-derived materials comprise a broad spectrum of bioactive compounds, including phenols, phenolic acids, flavonoids, tannins, terpenoids, alkaloids, and hydroxyl-containing constituents, which exhibit antimicrobial activity by disrupting the cell membrane integrity and inhibiting microbial growth.^8^

However, the efficacy of natural antioxidants and antimicrobial compounds in food systems is frequently constrained by factors such as low water solubility, chemical instability, sensitivity to heat and light, and limited bioavailability. To address these challenges, packaging films, coating materials, and delivery systems capable of providing controlled and sustained release of active compounds during storage have been developed.^9^ In this regard, nanotechnology-based approaches have emerged as innovative and effective strategies for the design of controlled release systems. Nanomaterials offer notable advantages in food preservation applications, including a high surface area, reduced required dosages, and the ability to enable controlled release of bioactive components.^10^

Within this framework, nanofibers have garnered considerable attention as promising materials for extending the shelf life of foods stored under refrigerated or ambient conditions, owing to their antimicrobial and antioxidant properties.^11,12^ The high surface area of nanofibers enables effective suppression of microbial growth, oxidative reactions, sensory deterioration, and certain physicochemical changes, while their low required application levels offer additional economic advantages.^13^

With the growing adoption of nanotechnological applications in the food industry, the use of nanofibers as carrier systems enabling a controlled release has become increasingly widespread. In particular, nanofibers fabricated from protein-based biopolymers have gained considerable importance in functional food preservation strategies due to their biodegradability, edibility, and capacity to enhance the stability of encapsulated bioactive compounds. Among these biopolymers, zein, a corn-derived protein with a Generally Recognized as Safe (GRAS) status, exhibits favorable film-forming properties and a hydrophobic structure, rendering it a suitable carrier matrix for bioactive substances such as phenolic compounds and essential oils.^14,15^

Accordingly, zein-based nanofiber systems developed to improve the efficacy of natural antioxidants and antimicrobial compounds have gained increasing relevance in applications involving meat products. In this study, the applicability of zein-based nanofibers incorporating bioactive components, namely, curcumin, gallic acid, and flaxseed oil, was evaluated in chicken meatballs, and the effects of these nanofiber coatings on the microbial and oxidative quality parameters of the product during storage were investigated in detail.

## 2 Material and Methods

### 2.1 Material

Chicken meat, flour, vegetable oil, and salt were procured from local markets in Van, Türkiye, while cold-pressed flaxseed oil was obtained immediately after pressing from a local producer to ensure freshness. All chemicals used in the study were of analytical grade and purchased from commercial suppliers (Sigma-Aldrich, Merck, Carlo Erba, Alfa Aesar, and Acros Organics), including zein, gallic acid, curcumin, Tween 20, bovine serum albumin (BSA), DPPH, Trolox, 2,4-dinitrophenylhydrazine (DNPH), thiobarbituric acid (TBA) reagent, guanidine hydrochloride, and microbiological media (MHA, MHB, PCA, and DRBC).

### 2.2 Methods

#### 2.2.1 Electrospinning Process and Application of Nanofibers on Chicken Meatballs

The zein spinning solution was prepared at a concentration of 20% (w/w) in 70% (v/v) ethanol and stirred for 24 h at room temperature by using a magnetic stirrer. For the preparation of curcumin-loaded zein nanofibers (ZNC), curcumin (0.0040 g) was added to 10 g of the zein solution and mixed for 1 h at room temperature. To obtain flaxseed oil- and curcumin-loaded nanofibers (ZNFC), flaxseed oil (0.75 g) and Tween 20 (0.10 g) were initially mixed, after which the 20% zein solution was added until a final weight of 10 g was achieved; curcumin (0.0040 g) was then incorporated, followed by stirring for 1 h. For the preparation of nanofibers co-loaded with flaxseed oil, curcumin, and gallic acid (ZNFCG), flaxseed oil (0.75 g), Tween 20 (0.10 g), and gallic acid (0.0150 g) were first mixed, the 20% zein solution was added to reach a total weight of 10 g, and curcumin (0.0040 g) was subsequently introduced; the resulting mixture was stirred for 1 h. All feed solutions were transferred to syringes prior to electrospinning.

Nanofibers were fabricated using an electrospinning setup comprising a high-voltage power supply, a syringe pump, and a flat collector (Fytronix ESP-900 Electrospinning System, Elazığ, Türkiye). Uniform and bead-free nanofibers were obtained under an applied voltage of 27 kV, a flow rate of 0.065 mL/min, and a needle-to-collector distance of 12 cm. The nanofibers were collected on an aluminum foil-covered metal collector.

Fresh chicken breast meat was minced, and meatball batter was formulated using 90% minced meat, 1.5% salt, 1% sunflower oil, and 7.5% flour. The meatballs were shaped to a standardized diameter of 62 mm and an average weight of 32 g. The prepared meatballs were subsequently wrapped with the electrospun nanofiber mats and stored under refrigerated conditions at 4±2 °C. Microbiological analyses, including total mesophilic aerobic bacteria (TMAB), total psychrophilic bacteria (TPB), and total yeast and mold (TYM) counts, as well as assessments of oxidative stability through thiobarbituric acid (TBA) values and protein carbonyl content, were conducted on days 1, 3, 5, 7, 10, 12, and 14 of storage. The coating application was performed at laboratory scale by manually wrapping the electrospun nanofiber mats around the meatball surface. In the present study, no separate mechanical tensile test, adhesion test, wettability analysis, water vapor or oxygen barrier measurement, or migration assay was conducted. Therefore, the practical performance of the coating was evaluated indirectly through its effects on microbiological growth, lipid oxidation, and protein oxidation during refrigerated storage. These material-level properties should be investigated in future studies before practical food-contact application.

#### 2.2.2 Morphological Characterization of Nanofibers

The morphologies of the fabricated nanofibers were examined using a scanning electron microscope (Supra 35VP; Carl Zeiss, Oberkochen, Jena, Germany) following sputter-coating with a thin layer of gold. The acquired SEM images were used to determine nanofiber diameters with the aid of ImageJ software.

#### 2.2.3 Molecular Characterization of Nanofibers (ATR-FTIR)

Molecular characterization of the nanofibers was carried out using attenuated total reflectance Fourier-transform infrared (ATR-FTIR) spectroscopy. Spectral measurements were obtained at 25 °C using an IR spectrometer (IR Affinity-1; Shimadzu Corporation) equipped with a single-reflection ATR accessory (MIRacle 10; Shimadzu Corporation) incorporating a diamond/ZnSe crystal. The spectra were recorded over the wavenumber range of 600–4000 cm^–1^ with a spectral resolution of 4 cm^–1^.

#### 2.2.4 In Vitro Controlled Release Analysis

The in vitro release behavior of bioactive compounds encapsulated within the nanofibers was assessed using the dialysis membrane method. Briefly, 50 mg of each nanofiber sample was dispersed in 10 mL of deionized water and transferred into dialysis membranes. A 50:50 (v/v) mixture of phosphate-buffered saline (PBS, pH 7.4) and ethanol was employed as the release medium. The dialysis bags were incubated at 37 °C with continuous agitation at 100 rpm for a period of 70 h. At predetermined time intervals, the amounts of released curcumin (420 nm), flaxseed oil (490 nm), and gallic acid (765 nm) were quantified spectrophotometrically. Cumulative release profiles were subsequently calculated as a function of time using the corresponding calibration curves.

#### 2.2.5 Encapsulation Efficiency

The encapsulation efficiency (EE) was determined by dispersing 50 mg of each nanofiber sample in 10 mL of an appropriate solvent. Methanol was used for the extraction of curcumin (CUR) and gallic acid (GA), whereas hexane was employed for flaxseed oil (FSO). For nanofiber formulations containing more than one bioactive compound, dispersions were prepared separately by using the corresponding solvents. The samples were agitated at 250 rpm for 1.5 h at room temperature in a shaking water bath and subsequently centrifuged at 4000 rpm for 30 min.

The EE of CUR and FSO was quantified spectrophotometrically at 420 and 490 nm, respectively, using a UV–Vis spectrophotometer (Aoelab, UV-1900PC). The EE of GA was calculated based on the total phenolic content (TPC) determined by the Folin–Ciocalteu method at 765 nm. Encapsulation efficiency was calculated using the following equation:

1 $$\mathrm{EE}\left(\%\right)=\frac{\mathrm{Total}\;\mathrm{bioactive}\;\mathrm{substance}-\mathrm{Bioactive}\;\mathrm{substance}\;\mathrm{in}\;\mathrm{the}\;\mathrm{filtrate}}{\mathrm{Total}\;\mathrm{bioactive}\;\mathrm{substance}}\times 100$$

#### 2.2.6 Antioxidant Properties of Nanofibers

The antioxidant properties of the nanofibers were evaluated through the determination of total phenolic content (TPC) and DPPH radical scavenging activity. For this purpose, nanofiber samples (50 mg) were extracted with methanol and homogenized in a temperature-controlled water bath. Following centrifugation, the obtained supernatants were used for antioxidant analyses. TPC was determined using the Folin–Ciocalteu method, while antioxidant activity was assessed by the DPPH radical scavenging assay. Quantification was performed using calibration curves prepared with gallic acid and Trolox standards, and the results were expressed as gallic acid equivalents (GAE) for TPC and Trolox equivalent antioxidant capacity (TEAC) for DPPH activity.^16^

#### 2.2.7 Antimicrobial Activity of Nanofibers

The antimicrobial activity of the nanofibers was assessed against *Escherichia coli* (ATCC 11303), *Salmonella typhimurium* (ATCC 13311), *Vibrio parahemolyticus* (ATCC 17802), *Pseudomonas aeruginosa* (ATCC 27853), *Staphylococcus aureus* (ATCC 25213), and *Bacillus cereus* (ATCC 14579) using disk diffusion and minimum inhibitory concentration (MIC) assays.

For the disk diffusion assay, bacterial suspensions were prepared from fresh cultures and adjusted to the 0.5 McFarland standard (approximately 1.5 × 10^8^ CFU/mL). Aliquots of the standardized suspensions were spread onto Mueller–Hinton agar (MHA) plates, and sterile disks impregnated with nanofiber extracts were placed on the agar surface. The plates were incubated at 37 °C for 24 h, after which inhibition zone diameters were measured in millimeters. Standard antibiotic disks (ampicillin, 10 μg; tetracycline, 30 μg) were used as positive controls.^17^

Minimum inhibitory concentration values were determined using the broth microdilution method in sterile 96-well microplates. Serial two-fold dilutions of the nanofiber extracts were prepared in Mueller–Hinton broth to obtain concentrations ranging from 0.20 to 0.025 mg/mL. Each well was inoculated with bacterial suspensions adjusted to the 0.5 McFarland standard and incubated at 37 °C for 24 h. After incubation, bacterial growth was evaluated by subculturing onto MHA plates, and the lowest concentration exhibiting complete inhibition of visible growth was recorded as the MIC value.^18^

#### 2.2.8 Microbial Analyses

To assess the effects of nanofiber coatings on microbial growth, total mesophilic aerobic bacteria (TMAB), total psychrophilic bacteria (TPB), and total yeast and mold (TYM) counts were determined for all samples. For microbiological analyses, 10 g of each sample was aseptically transferred into 90 mL of sterile 0.9% saline solution and homogenized using a stomacher (Stomacher 400, Seward, London, England). Serial decimal dilutions were then prepared in the range of 10^–1^ to 10^–6^.

Appropriate dilutions were plated onto the respective culture media and incubated under specific conditions. TMAB counts were determined after incubation at 35 °C for 24–48 h, TPB counts were assessed following incubation at 7 °C for 7–10 days, and TYM counts were evaluated after incubation at 25 °C for 72–120 h. Following incubation, microbial populations were enumerated and expressed as log CFU g^–1^.^19^

#### 2.2.9 TBA

Lipid oxidation was assessed using the thiobarbituric acid reactive substances (TBARS) method as described by Tarladgis et al. (1960),^20^ and the results were expressed as mg of malondialdehyde (MDA) per kg of sample.

#### 2.2.10 Carbonyl Content Analyses

Protein oxidation in chicken meatball samples during storage was evaluated by determining the total carbonyl content using the 2,4-dinitrophenylhydrazine (DNPH) derivatization method. Briefly, samples were homogenized in a potassium chloride (KCl) solution, and proteins were precipitated with trichloroacetic acid (TCA). The carbonyl groups present in the proteins were subsequently derivatized with DNPH. The resulting pellets were washed with an ethanol–ethyl acetate mixture, dissolved in guanidine hydrochloride, and the absorbance was measured spectrophotometrically. Protein concentration was determined using a bovine serum albumin (BSA) standard curve, and carbonyl content was expressed as nmol carbonyl per mg protein.^21^

#### 2.2.11 Statistical Evaluation

Group differences were evaluated using analysis of variance (ANOVA), which was conducted within a Python-based statistical analysis framework.^19,22,23^ The SciPy and statsmodels libraries were employed for the statistical analyses. These Python-based tools have been widely used in food science and food-related artificial intelligence studies, including data-driven analyses of allergen detection, food quality evaluation, and predictive modeling.^24^ Following ANOVA, post hoc multiple comparison tests were performed to identify specific differences among groups, and the Tukey’s honestly significant difference (HSD) test was applied for comprehensive pairwise comparisons.

## 3 Results and Discussion

### 3.1 Morphological Properties of Nanofibers (SEM)

The morphological characteristics of ZNC, ZNFC, and ZNFCG nanofibers produced by electrospinning were investigated by using scanning electron microscopy (SEM). Representative SEM micrographs of the nanofiber formulations are presented in Figure 1, revealing continuous, smooth, cylindrical, and bead-free fiber morphologies for all of the groups.

**Figure 1:**
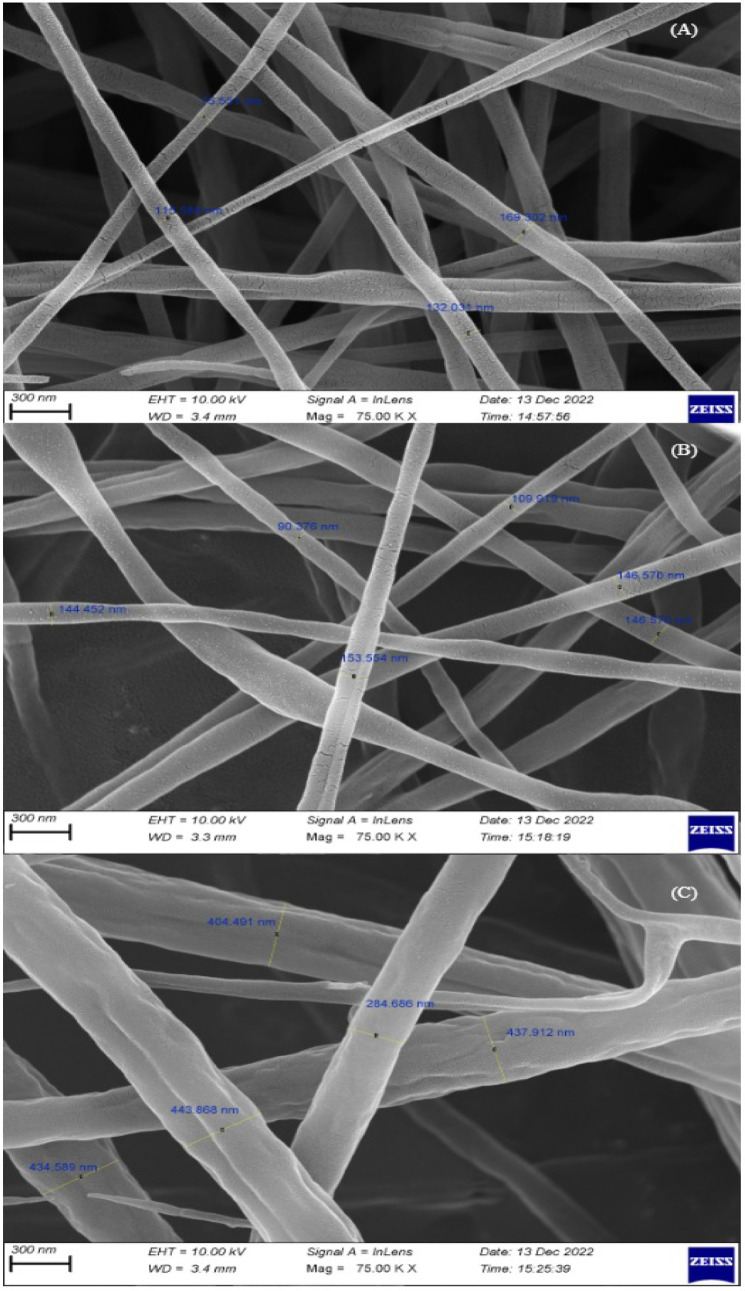
Scanning electron microscopy images of nanofibers: (A) ZNC, (B) ZNFC, and (C) ZNFCG.

Statistical analysis of fiber diameters revealed significant differences among the nanofiber formulations (p < 0.05). The smallest average fiber diameters were observed for the ZNFC (134.41 ± 21.12 nm) and ZNC (135.93 ± 48.91 nm) samples, with no statistically significant difference between these two groups. In both cases, minimum fiber diameters reached 90.37 and 75.55 nm, respectively, indicating that these nanofibers can be classified within the ultra-fine nanofiber range. In contrast, the ZNFCG formulation, which incorporated gallic acid in addition to curcumin and flaxseed oil, exhibited the largest average fiber diameter (338.54 ± 85.49 nm).

Previous studies have demonstrated that nanofiber diameter is strongly influenced by production parameters, polymer solution concentration, and the nature of bioactive compounds incorporated into the spinning solution.^25^ In particular, increases in solution viscosity are known to result in larger fiber diameters, and the addition of bioactive components may modify fiber morphology by altering both the viscosity and electrical conductivity of the electrospinning solution.^26–29^

In the present study, identical solvent systems, polymer concentrations, and electrospinning conditions were applied to all of the nanofiber formulations. Nevertheless, the pronounced increase in fiber diameter observed in the ZNFCG group indicates that the simultaneous incorporation of curcumin, flaxseed oil, and gallic acid exerts a substantial influence on the nanofiber morphology, likely through cumulative effects on the physicochemical properties of the spinning solution. From a structural perspective, the SEM images indicate that the electrospinning conditions were suitable for producing continuous nanofiber mats in all of the formulations. The absence of bead formation suggests sufficient chain entanglement in the zein solution and a stable electrospinning jet during fiber formation. The relatively narrow and uniform morphology observed in ZNC and ZNFC can be attributed to the compatibility of curcumin and flaxseed oil with the hydrophobic zein matrix. In contrast, the larger fiber diameter observed in ZNFCG suggests that the simultaneous incorporation of curcumin, flaxseed oil, and gallic acid altered the balance among viscosity, conductivity, and surface tension of the spinning solution. Gallic acid contains multiple hydroxyl groups and may interact with zein and curcumin through hydrogen bonding, thereby increasing the intermolecular interactions within the spinning solution. Such interactions can restrict jet stretching during electrospinning and lead to thicker fibers. Therefore, the morphological differences among the formulations support the conclusion that the incorporated bioactive compounds were not merely physically mixed with zein but also affected the structural organization of the nanofiber matrix.

### 3.2 Molecular Characterization of Nanofibers (ATR-FTIR)

Figure 2 shows the ATR-FTIR spectra of the nanofiber formulations.

**Figure 2:**
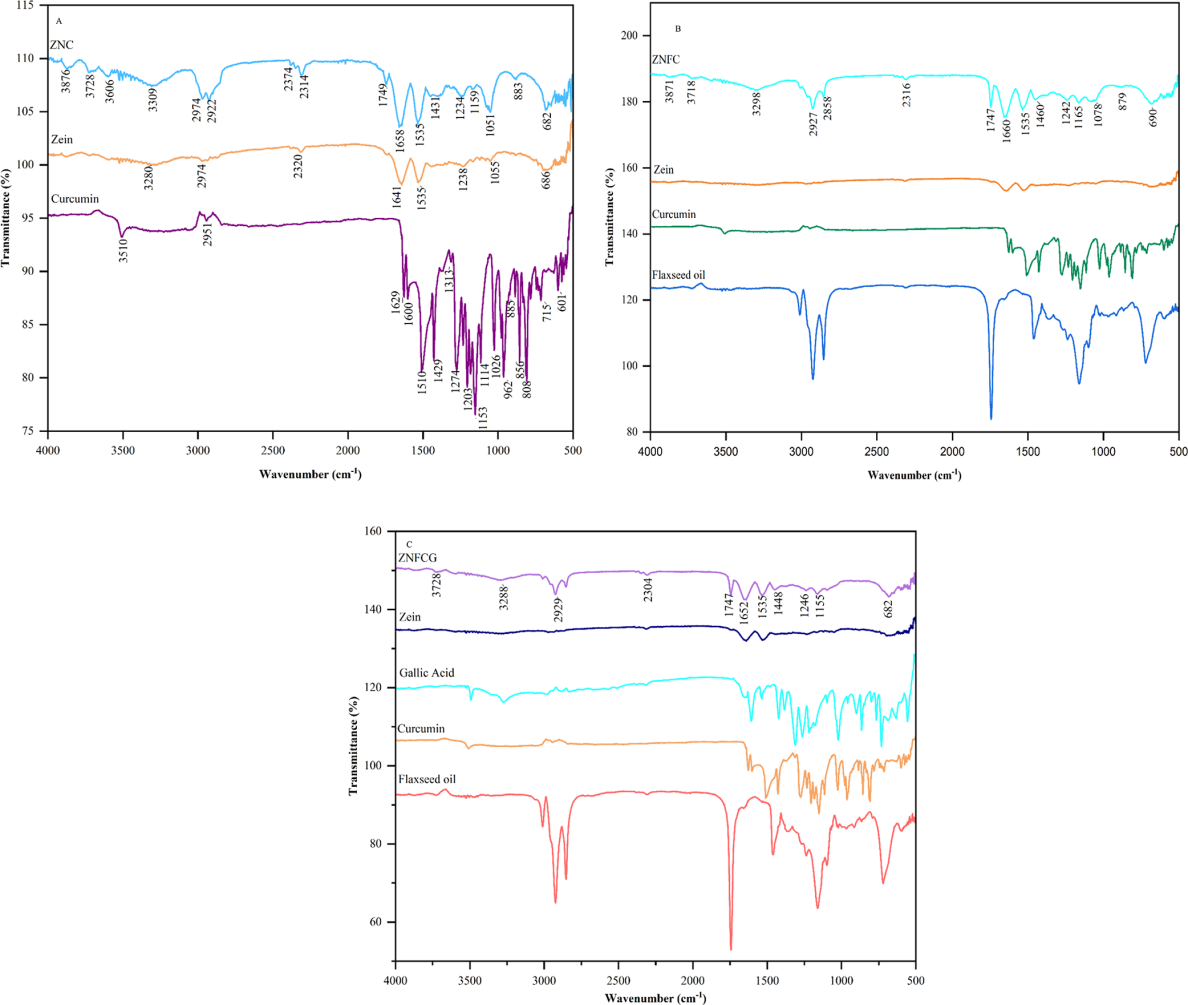
ATR-FTIR spectrum of (A) ZNC, (B) ZNFC, and (C) ZNFCG nanofibers.

ATR-FTIR spectroscopy was employed to confirm the incorporation of bioactive compounds into the zein matrix and to elucidate the molecular interactions within the electrospun nanofibers. The spectra of ZNC, ZNFC, and ZNFCG were analyzed in comparison with neat zein and the corresponding pure components. In all formulations, the characteristic absorption bands of the zein backbone were preserved, including the Amide I band at 1650–1660 cm^–1^, attributed to C=O stretching vibrations, and the Amide II band at 1530–1545 cm^–1^, arising from N–H bending coupled with C–N stretching. These features indicate that the zein protein backbone remained spectrally intact following electrospinning.^30^ The band observed at approximately 1260 cm^–1^ further confirmed the presence of Amide III groups,^31^ while the broad absorption band in the 3300–3800 cm^–1^ region was assigned to −OH and −NH stretching vibrations.^16,32^

Curcumin exhibited characteristic absorption bands at 1626 cm^–1^, corresponding to C=O/C=C stretching vibrations, and at 1429 cm^–1^, associated with phenolic C–O stretching, along with additional vibrational modes at 1314 and 1273 cm^–1^.^33–35^ In the ZNC formulation, shifts detected at 1510 cm^–1^ and 1026 cm^–1^, together with alterations in the O–H stretching region, indicated interactions between curcumin and zein functional groups, confirming the formation of hydrogen bonds within the nanofiber matrix.^36,37^ These spectral modifications are consistent with previously reported curcumin-induced changes in the secondary structure of zein.^31^

For formulations containing flaxseed oil (ZNFC and ZNFCG), absorption bands in the 2926–2854 cm^–1^ region were attributed to CH_2_ stretching vibrations of fatty acid chains, while the ester carbonyl band observed at 1745–1747 cm^–1^ confirmed the presence of triglyceride-type lipids within the nanofiber matrix.^38–41^ In the ZNFCG formulation, the broadened band spanning 3728–3288 cm^–1^ suggested extensive hydrogen bonding among zein amide groups and the hydroxyl functionalities of gallic acid and curcumin. Additional absorption bands in the 1246–1155 cm^–1^ range were assigned to C–O–C and C–O stretching vibrations, while characteristic low-frequency aromatic C–H bending bands were retained.^42^

Consequently, the ATR-FTIR results confirmed the preservation of the zein protein backbone and verified the successful spectral incorporation of curcumin and flaxseed oil into the ZNC, ZNFC, and ZNFCG nanofiber systems. The observed band shifts, changes in the −OH/–NH stretching region, and the presence of ester carbonyl markers collectively demonstrate the existence of matrix bioactive interactions within the electrospun nanofibers. The FTIR findings also provide insights into the possible interaction mechanisms governing the organization of the nanofiber matrix. The preservation of the amide I and amide II bands indicates that the zein backbone was maintained after electrospinning, suggesting that the process did not cause a major disruption to the protein structure. However, the shifts and broadening observed in the hydroxyl- and amide-related regions indicate the formation of secondary interactions between zein and the incorporated bioactive compounds. In particular, curcumin and gallic acid contain phenolic hydroxyl groups that can form hydrogen bonds with the carbonyl, amide, and amino groups of zein. The presence of flaxseed oil was supported by the lipid-associated C–H stretching bands and ester carbonyl signals, indicating the successful incorporation of the oil phase into the nanofiber structure. These molecular interactions may contribute to the high encapsulation efficiency and sustained-release behavior observed in the present study. Therefore, the FTIR results support not only the chemical presence of the bioactive compounds but also their interaction-based stabilization within the zein-nanofiber matrix.

### 3.3 In Vitro Controlled Release of Nanofibers

In this study, the in vitro controlled release behavior of curcumin from ZNC, ZNFC, and ZNFCG nanofibers was evaluated using the dialysis membrane method, and the time-dependent cumulative release profiles are presented in the corresponding figures (Figure 3). Controlled release systems are known to protect bioactive compounds against environmental and chemical degradation, thereby enhancing their stability, bioavailability, and functional efficacy.^43^ Moreover, zein-based carrier systems enable prolonged and regulated release through the coencapsulation of hydrophobic and hydrophilic compounds, facilitating the development of delivery systems capable of providing sustained bioactivity in food and pharmaceutical applications.^44,45^

**Figure 3:**
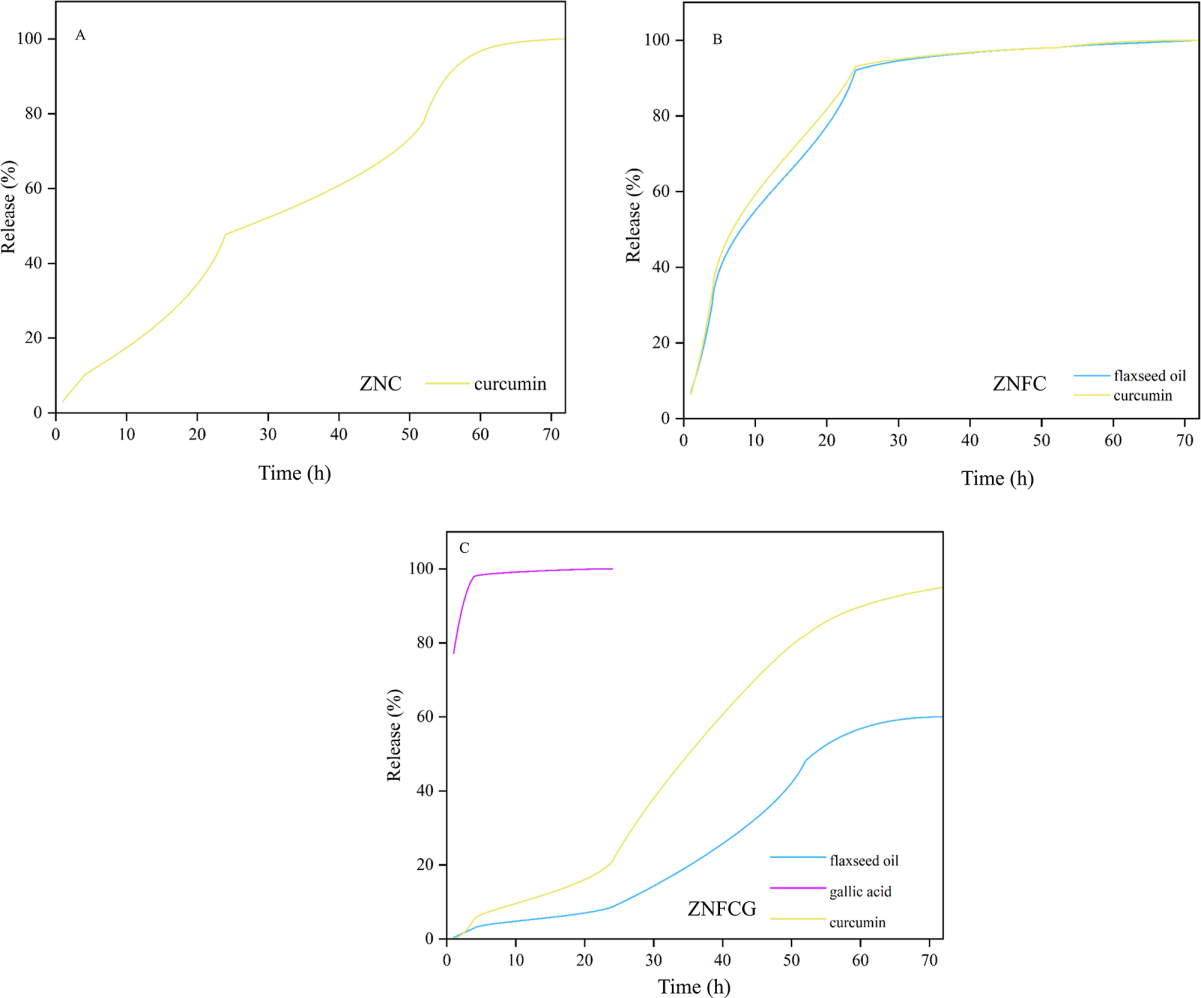
In vitro controlled-release profiles of the nanofibers: ZNC, ZNFC, and ZNFCG nanofibers, respectively.

For the ZNC nanofibers, the time-dependent release profile indicated that less than 20% of curcumin was released within the first 10 h, with a cumulative release reaching approximately 60% by 40 h and complete release achieved at 70 h. This release behavior reflects a sustained release profile comparable to that reported for alginate porous starch-based microemulsion gels, in which curcumin release became slower and more prolonged with increasing gel ratios.^46^

For the ZNFC nanofibers, the release profiles of curcumin and flaxseed oil were evaluated separately because both bioactive compounds were incorporated into the zein nanofiber matrix. Curcumin release showed a marked initial burst, with approximately 50% of the encapsulated curcumin released within the first 10 h. After this early phase, the release rate decreased and a slower, sustained release pattern continued until complete release was reached at 70 h. This biphasic release behavior is important for active food coating applications, since the initial burst may provide rapid bioactive availability at the coating–food interface, whereas the subsequent slower release phase may support prolonged functionality during refrigerated storage. Similar biphasic curcumin release behavior, consisting of rapid initial release followed by sustained release, has been reported for PLLA and PLLA–silk protein nanofibers.^47^

Flaxseed oil release from the ZNFC also followed a two-stage pattern. A rapid increase was observed during the early period, particularly within the first 10 h, and the release continued until approximately 25 h before approaching a slower plateau-like stage that extended to 70 h. Comparable release trends have been reported for oil-loaded nanofibrous systems, in which the release behavior is influenced by matrix composition, fiber morphology, and the physicochemical properties of the incorporated oil.^48,49^ In addition, the release characteristics of hydrophobic bioactives such as curcumin may vary depending on their distribution and interaction within structured matrices, as reported by Palla et al. (2022).^50^ Therefore, the ZNFC formulation can be described as a dual-bioactive nanofiber system providing both early-stage bioactive availability and subsequent sustained release, which may be advantageous for maintaining antioxidant and antimicrobial functionality in refrigerated food systems.

For the ZNFCG nanofibers, curcumin release initially proceeded at a relatively slow rate, reaching approximately 20% at 24 h, followed by a gradual and continuous increase, attaining about a 90% cumulative release by 70 h. This delayed and sustained release behavior indicates that the ternary incorporation of flaxseed oil, curcumin, and gallic acid into the zein nanofiber matrix influenced the release profile of the curcumin. A similar release behavior has been reported in SPI-succinic anhydride dextran-based nanogel systems, where He et al. (2022)^51^ observed a sustained curcumin release pattern from a protein-based delivery matrix. In addition, Mosallanezhad et al. (2025)^52^ reported prolonged curcumin release from core-shell nanofiber systems, further supporting the relevance of nanofibrous matrices for sustained bioactive release. Overall, the restrained initial release followed by a progressive release phase observed in the ZNFCG formulation suggests its suitability for food preservation applications requiring the prolonged availability of bioactive compounds during refrigerated storage.

Overall, the release profiles of curcumin-loaded nanofibers varied according to the formulation composition. While complete curcumin release was achieved by 70 h in both ZNC and ZNFC, the cumulative release in ZNFCG reached approximately 90% over the same period. These results demonstrate that curcumin release kinetics are strongly dependent on nanofiber composition and highlight controlled release behavior as a critical parameter in the rational design of nanofiber-based systems for food applications.

The in vitro release assay was conducted at 37 °C under controlled dialysis conditions to provide a standardized and comparative evaluation of formulation-dependent release behavior among ZNC, ZNFC, and ZNFCG. We acknowledge that these experimental conditions do not directly simulate refrigerated meat storage at 4 ± 2 °C. Temperature can markedly affect diffusion, polymer chain mobility, solvent penetration, and the release rate of encapsulated bioactive compounds. Therefore, the release profiles obtained at 37 °C should not be interpreted as direct evidence of release kinetics under refrigerated storage conditions. Instead, these data should be considered as comparative evidence showing the relative controlled-release potential of the nanofiber formulations. The actual preservation performance under refrigeration was evaluated independently through microbial growth, TBA, and protein carbonyl analyses during storage at 4 ± 2 °C. Future studies should specifically examine release kinetics at refrigerated temperatures and in real or simulated meat matrices.

### 3.4 Encapsulation Efficiency

The encapsulation efficiency of curcumin-loaded zein-based nanofibers was specifically evaluated for ZNC, ZNFC, and ZNFCG formulations. The encapsulation efficiency was calculated as the ratio of the amount of curcumin successfully incorporated into the nanofiber matrix to the total amount of curcumin initially used. The results demonstrated that curcumin was efficiently entrapped within the zein matrix at high levels in all of the nanofiber formulations examined.

For the nanofibers containing only curcumin (ZNC), the encapsulation efficiency was determined to be 96.32%. In the formulation co-loaded with curcumin and flaxseed oil (ZNFC), the encapsulation efficiency was calculated as 95.36%, whereas the formulation incorporating curcumin together with flaxseed oil and gallic acid (ZNFCG) exhibited a slightly lower, yet still high, encapsulation efficiency of 92.31%. Overall, these findings indicate that curcumin achieved encapsulation efficiencies exceeding 90% in all systems, whether used alone or in combination with other bioactive compounds.

Previous studies have reported that zein-based nanofibers exhibit a high loading and encapsulation capacity, particularly for hydrophobic bioactive compounds.^15,53^ Moreover, as electrospinning is conducted under ambient temperature and pressure conditions, it is especially advantageous for the encapsulation of heat-sensitive compounds such as curcumin, thereby preserving structural integrity during processing and enhancing stability and functionality in food-related applications.^54^ In agreement with these observations, Fereydouni et al. (2021)^55^ reported encapsulation efficiencies ranging from 91% to 96% for zein nanofibers incorporating nanocurcumin and concluded that zein represents an effective biopolymer for the delivery of lipophilic bioactive compounds.

The high encapsulation efficiencies obtained for ZNC, ZNFC, and ZNFCG in the present study highlight the suitability of zein-based nanofiber systems as effective biopolymer matrices for the incorporation and stabilization of curcumin.

### 3.5 Antioxidant Activity

In this study, the antioxidant capacity of zein-based nanofibers was evaluated by determining total phenolic content (TPC, mg GAE/g) and DPPH radical scavenging activity (mmol TEAC/g). Statistically significant differences were observed among the formulations depending on the type and combination of incorporated bioactive compounds (p < 0.05). Curcumin-loaded nanofibers (ZNC) exhibited a TPC of 71.58 ± 6.38 mg GAE/g and a DPPH value of 13.47 ± 0.61 mmol TEAC/g. When flaxseed oil was co-encapsulated with curcumin (ZNFC), TPC increased to 91.72 ± 4.87 mg GAE/g; however, the DPPH value remained relatively low at 10.08 ± 4.25 mmol TEAC/g, indicating that the higher phenolic content did not result in a proportional enhancement of radical scavenging activity in this formulation.

In contrast, the multibioactive nanofiber system (ZNFCG), incorporating curcumin, flaxseed oil, and gallic acid, exhibited the highest antioxidant performance, with a TPC of 117.11 ± 4.58 mg GAE/g and a DPPH value of 38.13 ± 0.99 mmol TEAC/g. These results demonstrate that the inclusion of gallic acid substantially enhanced radical scavenging capacity, and the DPPH activity of the ZNFCG formulation was comparable to that of gallic-acid-containing systems reported in the literature (p > 0.05). Although curcumin is recognized for its antioxidant potential due to the presence of phenolic −OH groups,^56^ the relatively low curcumin concentration used in this study (0.04%) may have limited its contribution to overall antioxidant activity. This observation is consistent with previous reports indicating that antioxidant performance in nanofibrous systems increases with higher curcumin loading levels.^34^

Collectively, these findings suggest that a direct linear relationship between TPC and DPPH activity may not always be observed, as TPC reflects the total concentration of phenolic compounds, whereas DPPH activity represents their effective radical-neutralizing capacity.^57,58^ Furthermore, factors such as the distribution, mobility, and binding or entrapment of phenolic compounds within the nanofiber matrix may significantly influence the antioxidant response measured by DPPH assays.^59^

The antioxidant activity results indicate that the effectiveness of zein-based nanofibers depends on the composition of incorporated bioactive compounds. While curcumin-containing nanofibers showed moderate antioxidant capacity, the inclusion of gallic acid together with curcumin and flaxseed oil significantly enhanced both total phenolic content and radical scavenging activity, highlighting the importance of multibioactive formulations for improved antioxidant performance.

### 3.6 Antimicrobial Activity

#### Inhibition Zone Assay

In this study, the antimicrobial performance of curcumin-containing zein-based nanofibers (ZNC, ZNFC, and ZNFCG) was evaluated by using the disk diffusion assay and compared with tetracycline (TE) and ampicillin (AMP) as positive controls (Table 1).

**Table 1:** Antimicrobial Effects of Nanofibers on Pathogenic Microorganisms (Zone Diameters in mm)[Table-fn tbl1fn1]

	*Staphylococcus aureus*	*Bacillus cereus*	*Escherichia coli*	*Salmonella typhimurium*	*Vibrio parahaemolyticus*	*Pseudomonas aeruginosa*
TE	22.00 ± 1.41^bA^	20.50 ± 2.12^bA^	21.00 ± 1.41^bA^	20.50 ± 0.70^bA^	29.50 ± 0.70^aA^	11.50 ± 0.70^cA^
AMP	13.50 ± 0.70^bB^	23.50 ± 2.12^aA^	12.50 ± 0.70^cB^	15.50 ± 0.70^bB^	13.50 ± 0.70^bcB^	0.00 ± 0.00^dC^
ZNC	15.50 ± 0.70^aB^	15.00 ± 0.00^abB^	11.50 ± 3.53^bcB^	10.50 ± 0.70^cC^	9.00 ± 0.00^cC^	10.00 ± 0.00^cB^
ZNFC	12.50 ± 3.53^abB^	14.50 ± 0.70^aB^	13.50 ± 2.12^abB^	0.00 ± 0.00^cD^	10.00 ± 0.00^bC^	0.00 ± 0.00^cC^
ZNFCG	15.00 ± 0.00^aB^	13.50 ± 0.70^aB^	12.00 ± 2.82^aB^	0.00 ± 0.00^bD^	14.50 ± 0.70^aB^	0.00 ± 0.00^bC^

i TE: tetracycline; AMP: ampicillin; ZNC: curcumin-loaded zein-based nanofiber; ZNFC: flaxseed oil and curcumin co-loaded zein-based nanofiber; ZNFCG: flaxseed oil, gallic acid, and curcumin co-loaded zein-based nanofiber. Values are expressed as mean ± standard deviation. Within the same column, means denoted by different letters differ significantly (p < 0.05). A–D indicate the antimicrobial effects of different nanofibers against the same pathogenic microorganism. a–d indicate differences in the antimicrobial efficacy of the same nanofiber against different pathogenic microorganisms.

The results demonstrated that all curcumin-containing nanofiber formulations exhibited microorganism-dependent inhibitory activity against both Gram-positive and Gram-negative foodborne pathogens; however, the extent and detectability of inhibition varied significantly depending on the specific bioactive composition of the nanofibers (p < 0.05).

Among Gram-positive bacteria, the most pronounced inhibitory effects were observed against *S. aureus*. Inhibition zone diameters of 15.50 ± 0.70 mm, 12.50 ± 3.53 mm, and 15.00 ± 0.00 mm were recorded for ZNC, ZNFC, and ZNFCG, respectively (Table 1, p <0.05), indicating that curcumin-based formulations, particularly those incorporating multiple bioactive compounds, can exert meaningful antibacterial effects. The antibacterial activity of curcumin against *S. aureus* has been associated in previous studies with the inhibition of sortase A, disruption of FtsZ-mediated cell division, and weakening of peptidoglycan integrity through phenolic interactions; these reported effects may help explain the relatively higher inhibition zones observed for ZNC and ZNFCG in the present study.^60–62^ In the case of *B. cereus*, comparable inhibition zones were observed for all curcumin-containing formulations (ZNC: 15.00 ± 0.00 mm; ZNFC: 14.50 ± 0.70 mm; ZNFCG: 13.50 ± 0.70 mm), suggesting similar antibacterial effectiveness against this Gram-positive pathogen.

For Gram-negative bacteria, a more selective inhibition profile was observed. All three curcumin-containing nanofibers produced measurable inhibition against *E. coli*, with inhibition zones ranging from 11.50 ± 3.53 mm to 13.50 ± 2.12 mm. In contrast, *S. typhimurium* was inhibited only by ZNC (10.50 ± 0.70 mm), while no inhibition was detected for ZNFC and ZNFCG (0.00 mm; p < 0.05). A similar pattern was observed for *P. aeruginosa*, where inhibition was detected exclusively for ZNC (10.00 ± 0.00 mm), whereas ZNFC and ZNFCG showed no measurable activity. For *V. parahaemolyticus*, clearer differentiation among formulations was evident: ZNC and ZNFC produced inhibition zones of 9.00 ± 0.00 mm and 10.00 ± 0.00 mm, respectively, while ZNFCG exhibited a significantly higher inhibition zone (14.50 ± 0.70 mm; p < 0.05). The reduced susceptibility commonly observed in Gram-negative bacteria is generally attributed to the presence of an outer membrane, restricted permeability through porins, and the structural complexity of the cell envelope, all of which can limit the penetration of antimicrobial agents.^63–66^ Accordingly, the absence of inhibition against *S. typhimurium* and *P. aeruginosa* in ZNFC and ZNFCG may reflect formulation-dependent dominance of these barrier effects, whereas the retained activity against *E. coli* and *V. parahaemolyticus* may be associated with interspecies differences in membrane composition and permeability.

In comparison with positive controls, TE exhibited strong inhibitory activity against all tested pathogens, while AMP showed pronounced effectiveness, particularly against *B. cereus*, but no inhibition against *P. aeruginosa* (Table 1). Although the inhibition zones produced by curcumin-containing nanofibers were generally smaller than those of conventional antibiotics, their ability to exert statistically significant inhibition against several key foodborne pathogens, especially *S. aureus*, *B. cereus*, *E. coli*, and *V. Parahemolyticus* supports the functional potential of nanofiber-based delivery systems.

Notably, the nanofiber disks weighed approximately 400 μg, corresponding to extremely low embedded bioactive amounts (approximately 30 μg flaxseed oil, 0.6 μg gallic acid, and 0.016 μg curcumin per disk). The observation of measurable inhibition zones at such low dosages underscores the advantages of nanoscale carrier systems, including a high surface-to-volume ratio, enhanced accessibility of active sites, and an increased likelihood of contact between bioactive compounds and target microorganisms.^67^ This is in line with previous reports demonstrating the superior efficacy of nanoencapsulated bioactives compared to their bulk counterparts.^68^ Within this framework, the higher inhibition zone observed for ZNFCG against *V. parahaemolyticus* (14.50 ± 0.70 mm) suggests that carrying multiple bioactive compounds within the nanofiber matrix may strengthen the antimicrobial response under specific conditions.

#### 3.6.2 Minimum Inhibitory Concentration (MIC)

The minimum inhibitory concentration (MIC) values of the curcumin-containing zein-based nanofiber formulations (ZNC, ZNFC, and ZNFCG) were determined using the broth microdilution method. The results revealed that the inhibitory efficacy against pathogenic microorganisms varied depending on the specific bioactive composition incorporated into the zein matrix. For *S. aureus*, the MIC was determined to be 0.1 mg/mL for both ZNC and ZNFC, whereas the MIC for ZNFCG exceeded 0.2 mg/mL. This outcome indicates that curcumin-containing formulations can effectively inhibit *S. aureus*; however, the simultaneous incorporation of three bioactive compounds does not necessarily enhance the antibacterial activity under all conditions. Previous studies have reported that the antibacterial activity of curcumin may be associated with effects such as disruption of membrane integrity, inhibition of biofilm formation, and induction of oxidative stress.^69,70^ In contrast, the higher MIC observed for ZNFCG may be associated with competitive localization of bioactives within the zein matrix or potential antagonistic interactions, as phenolic compounds may also exhibit pro-oxidant behavior under certain conditions.^71^

Against *B. cereus*, MIC values were 0.1 mg/mL for ZNFC and ZNFCG, and 0.2 mg/mL for ZNC, indicating that the incorporation of flaxseed oil in ZNFC and the ternary bioactive composition in ZNFCG enabled inhibition at lower concentrations compared to curcumin alone. Unsaturated fatty acids present in flaxseed oil, particularly linolenic acid, have been reported to exert antimicrobial effects by increasing membrane permeability and compromising bacterial structural integrity.^8^ Accordingly, the reduced MIC values observed for ZNFC and ZNFCG are consistent with a synergistic contribution of membrane-targeting effects from the oil phase in conjunction with the multitarget antibacterial activity of curcumin.

For the Gram-negative bacterium *E. coli*, the MIC decreased to 0.05 mg/mL for both ZNC and ZNFCG, whereas ZNFC exhibited an MIC value exceeding 0.2 mg/mL. This pronounced divergence highlights that even within the same carrier polymer, the combination of bioactive compounds critically influences antibacterial efficacy against Gram-negative bacteria. The outer membrane and lipopolysaccharide layer of Gram-negative bacteria act as significant permeability barriers that can restrict the diffusion of phenolic and lipophilic compounds. Nevertheless, the reduced MIC observed for ZNFCG may be attributed to the ability of gallic acid to interact electrostatically with the bacterial cell envelope and disrupt membrane integrity through reactive-oxygen-species-mediated pathways.^72^ Similarly, the low MIC value obtained for ZNC can be explained by the well-documented multimodal antibacterial action of curcumin, including damage to cell wall and membrane structures, suppression of DNA replication and gene expression, and induction of oxidative stress.^70^ In contrast, the limited activity of ZNFC against *E. coli* suggests that the combined presence of curcumin and flaxseed oil alone may be insufficient to overcome the permeability barrier, potentially due to formulation-dependent constraints on bioactive accessibility.

For *V. paraheamolyticus*, MIC values were 0.2 mg/mL for ZNC and ZNFCG, whereas ZNFC again exhibited an MIC exceeding 0.2 mg/mL. This pattern indicates that ZNC and ZNFCG were able to exert inhibitory effects to a certain extent against this Gram-negative pathogen, whereas ZNFC did not achieve inhibitory concentrations under the tested conditions. This trend parallels the observations for *E. coli*, suggesting that ZNFCG may offer improved performance against specific Gram-negative targets; however, such advantages are not uniformly expressed across all species.

The MIC results demonstrate that antimicrobial efficacy is governed not only by the presence of bioactive compounds but also by their type, combination, and spatial distribution within the nanofiber matrix. Among the curcumin-containing formulations, ZNC emerged as a particularly effective system with low MIC values against *S. aureus* and *E. coli*, while ZNFC showed enhanced activity primarily against *S. aureus* and *B. cereus* but limited efficacy against Gram-negative bacteria. ZNFCG exhibited low MIC values against *B. cereus* and *E. coli*; however, it did not provide improved inhibition against *S. aureus*. Collectively, these findings indicate that ternary bioactive incorporation does not necessarily result in universal synergistic effects and may, in some cases, reduce antibacterial efficacy due to matrix-level competition, antagonistic interactions, or pro-oxidant behavior of phenolic compounds.^73^ Accordingly, rational selection and optimization of bioactive combinations, taking into account target-specific cell wall and membrane characteristics, are essential for the effective design of zein-based antimicrobial nanofiber systems.

### 3.7 Microbial Growth

To evaluate the microbiological stability of chicken meatballs during refrigerated storage, total mesophilic aerobic bacteria (TMAB), total psychrophilic bacteria (TPB), and total yeast and mold (TYM) counts were monitored in both uncoated control samples and samples coated with curcumin-containing zein nanofibers. For clarity and consistency with the chicken meatball matrix, sample codes are denoted using the suffix “CM” (chicken meatball) (Table 2). The effect of neat zein nanofiber coating on chicken meatballs was previously evaluated in detail by our research group. In the aforementioned study, neat zein nanofibers (ZN), flaxseed oil-loaded zein nanofibers (ZNF), gallic acid-loaded zein nanofibers (ZNG), and flaxseed oil–gallic acid-loaded zein nanofibers (ZNFG) were applied to chicken meatballs, and their effects on microbial growth and lipid oxidation during refrigerated storage were investigated.^1^ Therefore, the present study, unlike the previous work, specifically focused on curcumin-containing zein nanofiber coatings and their multibioactive combinations with flaxseed oil and gallic acid in terms of the microbiological and oxidative quality of chicken meatballs.

**Table 2:** Changes in Microbial Quality Chicken Meatballs during Storage (log CFU/g)[Table-fn tbl2fn1]

Storage Period	Sample	TMAB	TPB	TYM
Initial		3.12 ± 0.05	3.70 ± 0.04	2.34 ± 0.20
1st day	Control	4.89 ± 0.06^aF^	3.87 ± 0.13^bF^	2.52 ± 0.21^aE^
	ZNC-CM	3.82 ± 0.56^bF^	3.84 ± 0.07^bG^	2.10 ± 0.14^bE^
	ZNFC-CM	3.74 ± 0.39^bF^	3.39 ± 0.27^cE^	2.06 ± 0.09^bC^
	ZNFCG-CM	4.00 ± 0.07^bC^	4.28 ± 0.02^aE^	2.10 ± 0.14^bD^
3rd day	Control	6.16 ± 0.11^aE^	5.18 ± 0.09^aE^	3.13 ± 0.20^aD^
	ZNC-CM	4.36 ± 0.48^bE^	4.80 ± 0.13^bF^	0 ± 0^cF^
	ZNFC-CM	3.87 ± 0.58^bcF^	4.52 ± 0.14^bD^	2.37 ± 0.26^bC^
	ZNFCG-CM	3.40 ± 0.08^cD^	3.93 ± 0.34^cF^	0 ± 0^cE^
5th day	Control	6.36 ± 0.39^aDE^	7.24 ± 0.01^aC^	3.19 ± 0.22^bcD^
	ZNC-CM	4.94 ± 0.45^bCD^	6.16 ± 0.08^bE^	3.03 ± 0.04^cD^
	ZNFC-CM	4.35 ± 0.10^cE^	5.66 ± 0.10^cC^	4.07 ± 0.06^aB^
	ZNFCG-CM	4.14 ± 0.07^cC^	4.96 ± 0.13^dC^	3.57 ± 0.50^bB^
7th day	Control	6.70 ± 0.29^aCD^	7.71 ± 0.28^aB^	4.75 ± 0.15^aC^
	ZNC-CM	4.55 ± 0.30^cDE^	6.84 ± 0.21^cC^	3.49 ± 0.35^bcC^
	ZNFC-CM	5.72 ± 0.22^bD^	7.36 ± 0.06^bB^	3.87 ± 0.62^bB^
	ZNFCG-CM	3.28 ± 0.21^dD^	5.31 ± 0.10^dC^	2.75 ± 0.66^cC^
10th day	Control	6.79 ± 0.08^aC^	6.81 ± 0.34^cD^	4.67 ± 0.54^aC^
	ZNC-CM	5.24 ± 0.18^bC^	7.35 ± 0.16^bB^	3.78 ± 0.22^bB^
	ZNFC-CM	6.67 ± 0.42^aD^	7.90 ± 0.47^aA^	4.97 ± 0.27^aA^
	ZNFCG-CM	4.26 ± 0.57^cC^	5.84 ± 0.28^dB^	2.15 ± 0.12^cD^
12th day	Control	7.59 ± 0.31^aB^	7.41 ± 0.08^aC^	5.42 ± 0.28^aB^
	ZNC-CM	6.38 ± 0.07^bB^	6.05 ± 0.33^bD^	3.15 ± 0.10^cD^
	ZNFC-CM	7.53 ± 0.13^aB^	7.46 ± 0.13^aB^	4.86 ± 0.20^bA^
	ZNFCG-CM	5.65 ± 0.12^cB^	5.45 ± 0.11^cC^	3.32 ± 0.15^cB^
14th day	Control	8.27 ± 0.23^aA^	8.37 ± 0.06^bA^	6.36 ± 0.06^aA^
	ZNC-CM	8.12 ± 0.07^aA^	8.77 ± 0.13^aA^	4.43 ± 0.15^cA^
	ZNFC-CM	8.27 ± 0.09^aA^	7.46 ± 0.09^cB^	5.08 ± 0.03^bA^
	ZNFCG-CM	7.89 ± 0.06^bA^	8.50 ± 0.06^bA^	4.13 ± 0.12^dA^

i Values are presented as mean ± standard deviation. Control group: uncoated chicken meatballs; ZNC-CM: chicken meatballs coated with curcumin-loaded zein nanofibers; ZNFC-CM: chicken meatballs coated with zein nanofibers co-loaded with flaxseed oil and curcumin; ZNFCG-CM: chicken meatballs coated with zein nanofibers co-loaded with flaxseed oil, gallic acid, and curcumin. Different lowercase letters (a–d) within the same storage day indicate significant differences among groups (p < 0.05). Different uppercase letters (A–F) within the same group indicate significant differences among storage days (p < 0.05).

#### 3.7.1 Total Mesophilic Aerobic Bacteria (TMAB)

The initial TMAB level (day 0) was 3.14 log CFU/g, which is consistent with values reported for chicken meatball products in the literature.^74,75^ During refrigerated storage, TMAB counts increased in all groups, and monitoring was continued until the commonly accepted microbiological spoilage limit of 6 log CFU/g for poultry products was exceeded.^76^ The uncoated control group (C-CM) reached this limit rapidly, attaining 6.11 log CFU/g on day 3, indicating an early loss of microbiological acceptability. In contrast, coatings containing curcumin-loaded zein nanofibers markedly delayed bacterial growth. On day 3, TMAB counts were 3.40 log CFU/g in ZNFCG-CM, 3.87 log CFU/g in ZNFC-CM, and 4.36 log CFU/g in ZNC-CM, demonstrating substantial inhibition relative to the control. This protective effect was sustained throughout storage. By day 10, the ZNFC-CM group exceeded the spoilage threshold (6.67 log CFU/g), whereas ZNC-CM remained below the limit (5.24 log CFU/g) and ZNFCG-CM exhibited the lowest TMAB level (4.26 log CFU/g). On day 12, ZNC-CM surpassed the limit (6.38 log CFU/g), while ZNFCG-CM continued to remain below the threshold (5.65 log CFU/g), reaching the limit only on day 14. Accordingly, based on the time required to reach 6 log CFU/g, the TMAB-defined refrigerated shelf life was extended from day 3 in the control samples to day 10 for ZNFC-CM, day 12 for ZNC-CM, and day 14 for ZNFCG-CM. These results indicate that the multibioactive ZNFCG formulation provided the most pronounced and sustained suppression of microbial growth. This enhanced performance is consistent with the high surface area and controlled release characteristics of nanofiber coatings, which enable prolonged antimicrobial activity,^77^ and is in agreement with previous studies reporting improved microbial stability in animal-based foods using bioactive nanofiber systems.^13^

#### 3.7.2 Total Psychrophilic Bacteria (TPB)

The initial TPB count was 3.70 log CFU/g (day 0). Because psychrophilic microorganisms dominate spoilage under cold storage, TPB was interpreted using the 6 log CFU/g acceptability criterion commonly applied to chilled meat systems.^78,79^ The C-CM group exceeded this threshold by day 5 (7.24 log CFU/g), confirming rapid cold-storage spoilage progression. Among coated samples, formulation-dependent differences were evident. On day 5, ZNC-CM exceeded the threshold (6.16 log CFU/g), whereas ZNFC-CM remained below it (5.66 log CFU/g) and ZNFCG-CM displayed the lowest TPB level (4.96 log CFU/g). By day 7, both ZNC-CM (6.84 log CFU/g) and ZNFC-CM (7.36 log CFU/g) surpassed the limit, while ZNFCG-CM remained below it (5.31 log CFU/g). Importantly, ZNFCG-CM stayed under 6 log CFU/g even on day 10 (5.84 log CFU/g) and reached the limit only on day 14, demonstrating prolonged control of refrigeration-adapted flora. The extended TPB stability observed in ZNFCG-CM supports the advantage of multicomponent bioactive systems under cold storage, where sustained surface availability and matrix–bioactive interactions contribute to persistent inhibition. Comparable psychrophilic suppression has been reported in nanofiber/nanocoating applications on poultry and seafood matrices.^78,80,81^

#### 3.7.3 Total Yeast and Mold (TYM)

The initial TYM load was 2.35 ± 0.21 log CFU/g (day 0), and storage time and formulation significantly affected fungal development (p < 0.05). The C-CM group showed continuous growth, reaching 6.31 log CFU/g at day 14, consistent with the susceptibility of protein-rich products to fungal proliferation during prolonged chilled storage.^82,83^ Curcumin-containing coatings yielded clear antifungal benefits by the end of storage. On day 14, TYM counts were 4.43 log CFU/g in ZNC-CM, 5.08 log CFU/g in ZNFC-CM, and 4.13 log CFU/g in ZNFCG-CM, indicating that the most effective suppression occurred in ZNFCG-CM. Previous studies have associated the antifungal activity of curcumin-based systems with effects such as inhibition of biofilm formation and filamentation, disruption of cellular energy processes, and increased membrane permeability.^84,85^ In addition, multicomponent natural antimicrobial systems have been shown to reduce yeast–mold development in poultry matrices, supporting the effectiveness of combined strategies.^82,86,87^

Consequently, microbial data consistently demonstrated that nanofiber coatings reduced bacterial and fungal proliferation during refrigerated storage, and the ZNFCG-CM formulation provided the most sustained protection across TMAB, TPB, and TYM metrics, indicating its suitability as an active coating strategy for shelf life extension in chicken meatballs.

### 3.8 TBA

Thiobarbituric acid (TBA) analysis is widely used to evaluate lipid oxidation in foods and is considered a key indicator of oxidative stability in meat products during refrigerated storage.^88,89^ In this study, chicken meatballs coated with zein-based nanofibers were stored at 4 °C for 14 days, and changes in TBA values (mg MDA/kg) were monitored (Table 3). On day 0, all groups showed a TBA value of 0.06 mg MDA/kg, consistent with the range reported for fresh meat products.^90^

**Table 3:** Changes in TBA and Carbonyl Content Chicken Meatballs during Storage[Table-fn tbl3fn1]

Storage Period	Sample	TBA (MDA/kg)	Carbonyl (nmol/mg)
Initial		0.06 ± 0.01	1.20 ± 0.01
1st day	Control	0.53 ± 0.01^aD^	2.15 ± 0.07^aG^
	ZNC-CM	0.53 ± 0.02^aA^	1.55 ± 0.02^bF^
	ZNFC-CM	0.62 ± 0.07^aA^	1.35 ± 0.07^cG^
	ZNFCG-CM	0.24 ± 0.01^bD^	1.15 ± 0.02^dG^
3rd day	Control	0.72 ± 0.01^aA^	3.85 ± 0.04^aF^
	ZNC-CM	0.12 ± 0.01^dE^	2.45 ± 0.01^bE^
	ZNFC-CM	0.29 ± 0.02^bD^	2.15 ± 0.26^cF^
	ZNFCG-CM	0.16 ± 0.00^cE^	1.55 ± 0.13^dF^
5th day	Control	0.64 ± 0.01^aB^	5.95 ± 0.03^aE^
	ZNC-CM	0.13 ± 0.01^dE^	3.55 ± 0.07^bD^
	ZNFC-CM	0.53 ± 0.02^bB^	3.25 ± 0.03^cE^
	ZNFCG-CM	0.29 ± 0.00^cC^	2.40 ± 0.14^dE^
7th day	Control	0.49 ± 0.01^bE^	11.85 ± 0.06^aB^
	ZNC-CM	0.43 ± 0.05^cB^	4.15 ± 0.01^bC^
	ZNFC-CM	0.52 ± 0.01^bB^	3.85 ± 0.02^cD^
	ZNFCG-CM	0.73 ± 0.01^aB^	3.05 ± 0.03^dD^
10th day	Control	0.56 ± 0.01^bC^	10.15 ± 0.02^aC^
	ZNC-CM	0.37 ± 0.01^dC^	2.85 ± 0.21^cE^
	ZNFC-CM	0.50 ± 0.01^cB^	4.85 ± 0.01^bC^
	ZNFCG-CM	0.77 ± 0.02^aA^	3.55 ± 0.01^cC^
12th day	Control	0.51 ± 0.01^aDE^	8.05 ± 0.03^aD^
	ZNC-CM	0.27 ± 0.01^cD^	6.75 ± 0.12^bB^
	ZNFC-CM	0.41 ± 0.01^bC^	5.45 ± 0.03^cB^
	ZNFCG-CM	0.14 ± 0.00^dE^	4.25 ± 0.02^dB^
14th day	Control	0.37 ± 0.01^aF^	13.55 ± 0.13^aA^
	ZNC-CM	0.23 ± 0.01^cD^	7.55 ± 0.22^bA^
	ZNFC-CM	0.33 ± 0.01^bD^	6.55 ± 0.02^cA^
	ZNFCG-CM	0.09 ± 0.00^dF^	4.75 ± 0.07^dA^

i Values are presented as mean ± standard deviation. Control group: uncoated chicken meatballs; ZNC-CM: chicken meatballs coated with curcumin-loaded zein nanofibers; ZNFC-CM: chicken meatballs coated with zein nanofibers co-loaded with flaxseed oil and curcumin; ZNFCG-CM: chicken meatballs coated with zein nanofibers co-loaded with flaxseed oil, gallic acid, and curcumin. Different lowercase letters (−d) within the same storage day indicate significant differences among groups (p < 0.05). Different uppercase letters (A–F) within the same group indicate significant differences among storage days (p < 0.05).

Throughout storage, fluctuations (increases/decreases) in TBA values were observed, yet none of the samples exceeded 1 mg of MDA/kg, which is commonly cited as the acceptability threshold for oxidative deterioration.^91^ The uncoated control (C-CM) increased rapidly to 0.53 mg of MDA/kg on day 1 and reached 0.72 mg of MDA/kg by day 3. In comparison, curcumin-containing coatings notably suppressed early MDA formation: on day 3, TBA values were 0.12 mg of MDA/kg (ZNC-CM) and 0.29 mg of MDA/kg (ZNFC-CM), while ZNFCG-CM remained at 0.16 mg of MDA/kg, indicating substantially improved oxidative stability relative to the control at the early storage stage.

Marked differences among the curcumin-based coatings were evident over time. ZNC-CM maintained relatively low TBA values during storage, reaching 0.23 mg of MDA/kg on day 14. ZNFC-CM also limited lipid oxidation with a final value of 0.33 mg MDA/kg on day 14. The most pronounced protection was observed for ZNFCG-CM, which achieved the lowest endpoint TBA value (0.09 mg of MDA/kg, day 14). The strong reduction in MDA accumulation observed in the curcumin-based systems is consistent with the reported radical-scavenging capability of curcumin and its ability to suppress chain lipid oxidation reactions.^92^ Moreover, the superior performance of ZNFCG-CM supports the notion that multicomponent antioxidant formulations can provide enhanced oxidative stabilization compared with single or binary systems, potentially through combined protective actions during storage.^59,93^

Consequently, the TBA findings indicate that curcumin-containing zein nanofiber coatings effectively restrained lipid oxidation in chicken meatballs during refrigerated storage, with the ZNFCG formulation showing the strongest protection, as reflected by the minimal MDA level at the end of storage. The observed fluctuations in TBA values across storage days are in agreement with reports suggesting that MDA formation may continue up to a certain point and can later decrease because of decomposition or secondary reactions.^94^

### 3.9 Carbonyl Content

Lipid and protein oxidation are major deterioration pathways in meat products, causing undesirable changes in color, flavor, odor, texture, and nutritional quality.^95^ Protein oxidation is commonly assessed through the formation of carbonyl compounds, which are regarded as key biomarkers of oxidative modification in muscle proteins.^3,96^ Reactive oxygen species (ROS) and lipid oxidation products can induce irreversible structural damage in proteins, leading to increased carbonyl formation and associated quality loss.^71,97^ In the present study, carbonyl content (nmol/mg) was monitored in chicken meatballs during refrigerated storage, and significant differences among treatments were observed (Table 3) (p < 0.05).

The initial carbonyl content of the raw material was 1.20 nmol/mg (day 0). During storage, the uncoated control (C-CM) showed a pronounced increase, reaching 13.55 nmol/mg by day 14, consistent with progressive oxidative stress in the absence of protective agents and with the potential involvement of ROS and lipid-derived reactive compounds in protein carbonylation.^10^ In contrast, curcumin-based nanofiber coatings substantially limited carbonyl accumulation over the same period. By day 14, carbonyl levels were reduced to 7.55 nmol/mg (ZNC-CM) and 6.55 nmol/mg (ZNFC-CM), while ZNFCG-CM exhibited the lowest value (4.75 nmol/mg), indicating the strongest suppression of protein oxidation.

Notably, the divergence among curcumin-based coatings was already apparent during midstorage. On day 7, carbonyl contents were 4.15 nmol/mg in ZNC-CM, 3.85 nmol/mg in ZNFC-CM, and 3.05 nmol/mg in ZNFCG-CM, whereas the control had increased to 11.85 nmol/mg in C-CM. The improved protection observed for ZNFCG-CM is consistent with previous reports indicating that phenolic-rich systems may reduce protein oxidation through antioxidant-related effects, including radical scavenging and metal-chelating activity, thereby limiting the formation of carbonyl derivatives in susceptible protein structures.^98^ Furthermore, polyphenolic compounds have been reported to slow thiol oxidation and reduce carbonyl formation in meat matrices, supporting the lower carbonyl levels observed in the multicomponent coating.^99^

As a result, these findings demonstrate that curcumin-containing zein nanofiber coatings mitigated protein oxidation in chicken meatballs during refrigerated storage, with ZNFCG-CM providing the greatest protection, as evidenced by the lowest final carbonyl content. This confirms that nanofiber-based coating systems can contribute not only to microbial control but also to the preservation of oxidative quality in meat products.

### 3.10 Practical Applicability, Food-Contact Safety Considerations, and Study Limitations

Although the present study demonstrates that bioactive-loaded zein nanofiber coatings can delay microbial proliferation and oxidative deterioration in chicken meatballs during refrigerated storage, several practical and safety-related limitations should be considered. Zein is a corn-derived protein widely used as a biopolymeric carrier in food-related applications, and the incorporated bioactive compounds, namely, curcumin, gallic acid, and flaxseed oil, are food-derived or food-associated compounds. However, the safety of the final nanofiber coating cannot be concluded solely on the basis of the food-compatible nature of its individual components. In the present study, cytotoxicity, cytocompatibility, migration behavior, and detailed food contact safety assessments were not performed. Therefore, the findings should be interpreted as evidence of preservative efficacy under laboratory-scale refrigerated storage conditions rather than as a complete toxicological or regulatory validation of the coating system.

The practical applicability of nanofiber coatings also depends on material-related properties such as coating thickness, mechanical integrity, adhesion to the meat surface, wettability, handling stability, and water vapor or oxygen barrier performance. These parameters were not directly measured in the present study. Instead, the functional performance of the coatings was evaluated indirectly by monitoring microbiological quality, lipid oxidation, and protein oxidation in coated chicken meatballs during storage at 4 ± 2 °C. The observed reductions in total mesophilic aerobic bacteria, psychrophilic bacteria, yeast–mold counts, TBA values, and carbonyl content indicate that the coatings remained functionally active during refrigerated storage. Nevertheless, further studies are required to quantify coating thickness, mechanical resistance, surface adhesion, wettability, barrier properties, and structural stability during storage.

Another limitation concerns the release analysis. The in vitro release assay was conducted at 37 °C under controlled dialysis conditions to compare the release behavior of the different nanofiber formulations. These conditions provide useful comparative information on formulation-dependent release profiles, but they do not directly represent refrigerated meat storage at 4 ± 2 °C. Therefore, the release results should be considered supportive evidence of controlled-release potential rather than direct proof of release kinetics under refrigerated storage conditions. Future studies should include release experiments at refrigerated temperatures and preferably in real or simulated meat matrices.

In addition, thermal characterization methods, such as differential scanning calorimetry or thermogravimetric analysis, were not performed in the present study. Therefore, no direct conclusions can be drawn regarding the thermal transition behavior or the thermal degradation profile of the developed nanofiber coatings. Future studies should include thermal analyses to better understand the processing stability, storage stability, and temperature sensitivity of bioactive-loaded zein nanofibers.

Overall, the present study should be regarded as a laboratory-scale investigation demonstrating the preservative potential of multibioactive zein nanofiber coatings for refrigerated chicken meatballs. Before practical or industrial application, additional validation studies addressing cytotoxicity, migration, food-contact safety, mechanical integrity, adhesion, wettability, barrier performance, refrigeration release behavior, and thermal properties are necessary.

## 4 Conclusion

Electrospun zein nanofibers co-loaded with curcumin, flaxseed oil, and gallic acid provided a clear preservation advantage for chicken meatballs during refrigerated storage. Among all formulations, ZNFCG showed the most consistent and strongest protection, extending microbiological acceptability from day 3 (control) to day 14, as indicated by the delayed rise of total mesophilic aerobic bacteria to the 6 log CFU/g threshold. The same trend was reflected in psychrophilic bacteria and yeast–mold counts, which remained lower in coated samples, with ZNFCG generally exhibiting the lowest loads at late storage.

Beyond microbial control, the coatings also restrained oxidative quality loss. ZNFCG achieved the lowest protein oxidation at the end of storage (carbonyl content), confirming superior protection of meat proteins under refrigeration. Taken together with the release behavior of the bioactives from the nanofiber matrix, these findings support a sustained “active surface” effect, helping ZNFCG maintain functionality throughout storage rather than providing only a short-lived initial activity. Overall, ZNFCG zein nanofibers represent a practical active-coating strategy to extend refrigerated shelf life up to 14 days while simultaneously limiting microbial growth and oxidative deterioration.
